# Evaluating Growth Response, Hemo‐Biochemical Parameters, and Slaughter Performances of Rabbits Fed *Moringa oleifera* Leaf Meal–Based Diets: A Systemic Review and Meta‐Analysis

**DOI:** 10.1002/fsn3.70738

**Published:** 2025-09-12

**Authors:** Freddy Manyeula, Nthabiseng Amenda Sebola, Monnye Mabelebele

**Affiliations:** ^1^ Department of Animal Sciences, Faculty of Animal and Veterinary Sciences Botswana University of Agriculture and Natural Resources Gaborone Botswana; ^2^ Department of Agriculture and Animal Health, College of Agriculture and Environmental Sciences University of South Africa Pretoria South Africa

**Keywords:** blood, feed ingredients, feed intake, rabbit production, slaughter performance, weight gain

## Abstract

*Moringa oleifera* leaf is known for its many nutritional and nutraceutical properties, which are essential for the growth performance and well‐being of the animal. So, in this review, we aim to use meta‐analytical procedures to resolve uncertainty, identify knowledge gaps, and create new insights using published data on the effects of *M. oleifera* leaf meal (MOLM) on growth responses, hemo‐biochemical parameters, and slaughter performance of rabbits. Google and electronic databases were employed to search for studies on feeding rabbit MOLM‐based diet (MOLMBD), and the data generated were analyzed using OpenMEE software. Sources of heterogeneity were evaluated using rabbit breed, inclusion levels, study periods, and sex. Results reveal that rabbits fed MOLMBD had similar feed intake (standardized mean difference (SMD) = 0.07; 95% confidence interval (CI): −0.19, 0.05; *p* = 0.001) with higher average daily gain (SMD = 0.86; 95% CI: 0.63; 1.09; *p* = 0.001) along with an improved feed conversion ratio (FCR) (SMD = −0.90; 95% CI: −1.11, −0.69; *p* = 0.001). Additionally, MOLM improved white and red blood cells, while lymphocytes, mean corpuscular volume, and mean corpuscular hemoglobin significantly reduced. Blood plasma and slaughter performance were affected positively by a MOLMBD when fed to rabbits. The study revealed that the inclusion levels, breeds, sex, and feeding duration were responsible for inconsistent results among studies. We conclude that feeding rabbits MOLMBD promotes weight gain and FCR and did not significantly affect feed intake (FI). Further research on the production of *M. oleifera* and its utility on animal feeds is needed.

## Graphical abstract

This flow diagram showed a systematic review to determine the impact of feeding rabbit *Moringa oleifera* leaf meal‐based diet (MOLMB) on hemo‐biochemical parameters and slaughter performances. Results showed that feeding rabbit MOLMB had similar feed intake with higher average daily gain along with improved feed conversion, blood cell and plasma, and slaughter performance. These findings emphasize the importance of MOLMB as an alternative ingredient in rabbit diets.

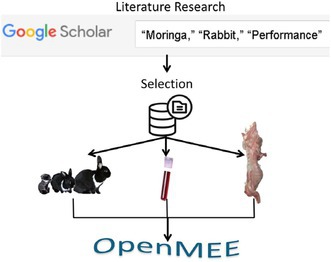

## 1 Introduction

Rabbits are a micro‐livestock producing about 47 kg of meat per doe per year, which could contribute to meeting the nutrition and food security of the country. It is mostly rural communities that eat rabbits to meet their daily nutritional requirements (Siddiqui et al. 2023). Rabbit meat is rich in essential amino acids, polyunsaturated fatty acids, vitamins, and minerals and is low in cholesterol (Elazab et al. 2022), which is regarded as healthy as far as consumers are concerned. However, the high cost of soybean meal in commercial rabbit diets limits the potential of rabbit farms to contribute to the nutrition and food security of South Africa (Zwoliński et al. 2017). Due to the intensification of rabbit farming, feed costs can reach up to 70% of total costs (Gidenne et al. 2017), a figure that is greatly influencing feed conversion ratio (FCR). The latter scenario, along with the fact that the European Commission banned the use of antibiotics as growth agents in 2006 (Abou‐Zeid et al. 2015), prompted the researchers to opt for other plant leaf meal like *Moringa oleifera* leaf meal (MOLM) as a feed additive—one that has shown the ability to increase growth rate and feed efficiency in rabbit production, even at a reduced feed intake (FI).

*Moringa oleifera* (MO) is a deciduous, evergreen tree of the Moringaceae family, and it can do well in a variety of soil types. It also grows well in hot and humid, as well as dry tropical and subtropical climates (Ferreira et al. 2008). MOLM is normally incorporated into the diets of monogastric animals due to its nutritional and medicinal properties (Laptev et al. 2021). Makkar and Becker (1996) reported high nutritional and nutraceutical properties of MOLM, and these are known to promote growth performance (Mankga et al. 2022) in rabbits. The proximate analysis of MOLM was reported by Moyo et al. (2011) indicating 30.29% crude protein, 5.49% crude fiber, 11.40% neutral detergent fiber, 8.49% acid detergent fiber, and 7.64% ash. *Moringa oleifera* is known to contain well‐balanced amino acids, vitamins (A, B, C, E, and K) (Gopalakrishnan et al. 2016), and phytochemicals such as beta‐carotene, flavonoids, alkaloids, chlorogenic acid, and phenolic acids, which are known to have antioxidant, anti‐inflammatory, antiviral, antifungal, and antimicrobial properties (Falowo et al. 2018). Also, these compounds have immune‐stimulating and immune‐modulating effects in humans and animals (Kou et al. 2018). Mbikay (2012) also reported high amounts of monounsaturated and polyunsaturated fatty acids in MOLM, which are known to influence cell and tissue metabolism and function. Besides these important bioactive compounds, *M. oleifera* contains anti‐nutritional factors (ANFs), such as condensed tannins (0.33 AU550 nm/10 mg) and gallic acids (Sebola et al. 2019); Stevens et al. (2015) reported phytates (2.23%), oxalates (1.42%), saponins (2.06), and alkaloids (1.56%). These compounds interfere with mineral and protein utilization in the gastrointestinal tract of the animal, while high fiber levels increase the relative weight of cecal contents and their residence time (Liu et al. 2022) in monogastric animals. Interestingly, the growth rate of rabbits was found to be high when fed a diet containing a high amount of cellulose and alfalfa supplemented group compared to those fed lignin (Chiou et al. 1998). Moreover, further research was conducted to study the effect of MOLM on the growth performance traits of rabbits elsewhere, but the results remained inconclusive. Some studies (Mankga et al. 2022; Aljohani and Abduljawad 2018; El‐kashef 2022) reported increases in rabbit growth performance parameters, and others (Olatunji et al. 2016; Ogunlade et al. 2019) indicate a lack of significant effects in rabbits fed MOLM. On the contrary, Abiodun and Olubisi (2017) reported a reduction in growth performance parameters. These published works do not provide any solid scientific basis for defining growth performances because the published results are sometimes contradictory and inconclusive. However, we believe that this matter can be solved by meta‐analysis, and the findings could help farmers, researchers, or a broader audience make an informed decision on the effects of MOLM on the growth performance of rabbits. Meta‐analysis employs the data available and fits it to the experimental diversity, thereby increasing the sample size and exposing possible differences that small populations would not be able to show (Remus et al. 2014). Thus, this study provides a summary of the current literature available on the use of MOLM feeding in rabbits and sets out to predict the FI, weight gain, and FCR response to MOLM. We hypothesized that meta‐analysis would provide a possible conclusion as to the effects of MOLM use on rabbit growth performance. However, this meta‐analysis tries to answer the research question that “Does feeding rabbits diets containing MOLM have significant overall effects on growth performance, hemo‐biochemical parameters, and slaughter performance?”

## 2 Materials and Methods

### 2.1 Search Strategy and Inclusion Criteria

**FIGURE 1:**
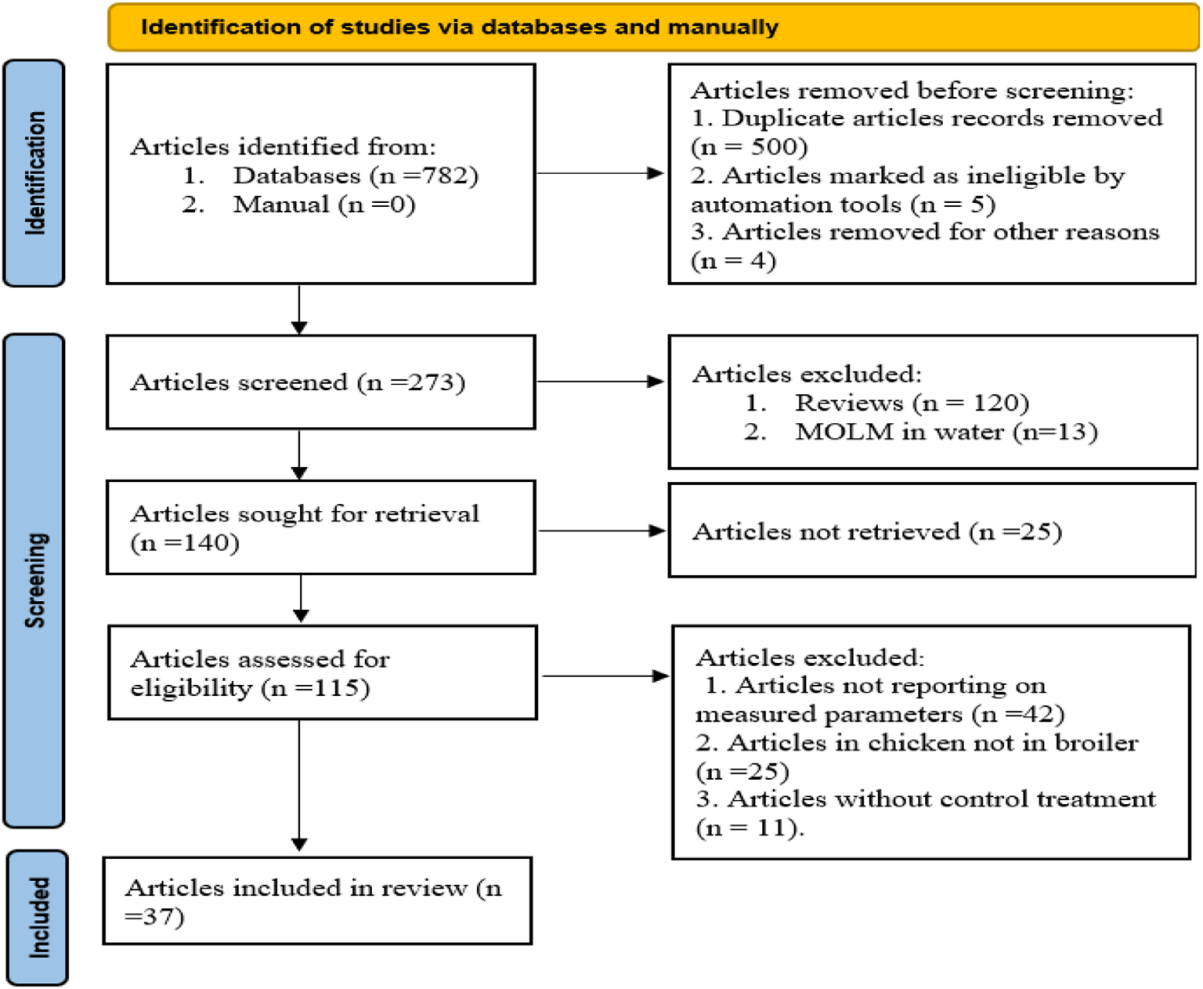
Literature search and selection process following the PRISMA procedure.

Predefined scientific databases, such as Google Scholar, Scopus, and PubMed, were used, along with the Google search engine, to search the literature. The database searches used the terms *M. oleifera* , growth performance, blood, and rabbits (Table 1), but were not restricted to many keywords. A maximum of 782 publications was found, and all duplicate publications were identified and removed. Once the duplicate publications had been removed, the databases were recorded in Microsoft Excel 2003. The data entered included the full name of the first author in each case to identify the publications. During the literature search, the title and abstract of each publication were used to exclude irrelevant publications. All relevant publications were read, and the growth performance parameters FI, weight gain, and FCR were recorded for possible classification of eligibility. For further selection, the following inclusion criteria were applied: (1) publications that compared different MOLM inclusion with the control diet, (2) publications with detailed descriptions of a chemical analysis of the diet, (3) any publications that reported six of the following parameters: rabbits, FI, weight gain, FCR, hemo‐biochemical parameters, and MOLM, and that were published in English. Studies where enzymes were incorporated with MOLM were excluded. Only peer‐reviewed articles were considered. A total of 37 articles were considered eligible for this meta‐analysis, using the selection process shown in Figure 1.

**TABLE 1:** Search strategies for Google Scholar, Scopus, Web of Science, and PubMed databases.

Database	Search	Studies identified
Google Scholar	(“*Moringa oleifera leaves”* OR (“rabbits” AND “growth performance”) OR “blood”)	700
Scopus	(“*Moringa oleifera leaves”* OR (“rabbits” AND “growth performance”) OR “blood”)	23
Web of Science	(“*Moringa oleifera leaves”* OR (“rabbits” AND “growth performance”) OR “blood”)	48
PubMed	(“*Moringa oleifera leaves”* OR (“rabbits” AND “growth performance”) OR “blood”)	11

### 2.2 Data Synthesis

Data were extracted (Table 1) on study identification (author surname, year of publication), study country (South Africa, Nigeria, India, Egypt, China, Indonesia, Saudi Arabia, and Cameroon), continent (Africa and Asia), growth performance, hematology, blood chemistry, and carcass traits. Data were also collected on MOLM inclusion levels (0%–100%), breed of rabbit (New Zealand, Chinchilla, Not stated, mixed, and Monshtoher breeds), sex (male, unsexed, and unknown) and study periods (> 8 to < 20 weeks) as shown in Table 2. Where authors whose studies were used for the analysis did not state the breed and sex of rabbits used in their studies, these rabbits were named “unknown”.

**TABLE 2:** Characteristics of eligible studies used for the analysis.

Studies	Country	Form	Studied moderators			Sex	Outcomes
			Inclusion	Breed	SD		
Ewuola et al. (2012)	Nigeria	Leaves	0, 5, 10, 15	Crossbreed	10	Unknown	GP
Dougnon et al. (2012)	Benin	Leaves	0, 10, 15	Crossbreed	10	Unknown	GP, CT
Yakubu et al. (2013)	Nigeria	Leaves	0, 25, 50, 75, 100	Mixed	8	Unsexed	GP
El‐Badawi et al. (2014)	Egypt	Leaves	0, 0.15, 0.30	Nzealand	8	Male	GP, CT
Olatunji et al. (2015)	Nigeria	Leaves	0, 5,10, 15	Mixed	8	Unsexed	H, BC
Abubakar et al. (2015)	Nigeria	Leaves	0, 15, 30, 45	Mongrel	8	Unknown	CT
Jiwuba et al. (2016)	Nigeria	Leaves	0, 10, 20, 30	Mixed	7	Unknown	H, BC
Abiodun and Olubisi (2017)	Nigeria	Leaves	0, 2.5,5, 7.5	Crossbreed	> 12	Male	GP, BC
Helal et al. (2017)	Egypt	Leaves	0, 1	Nzealand	10	Male	GP, CT
Ayo‐Ajasa et al. (2017)	Nigeria	Leaves	0, 15, 30, 45	Not stated	10	Unsexed	GP
Bolarin et al. (2017)	Nigeria	Leaves	0, 15, 30	Nzealand	12	Unsexed	GP, H, BC
Gomaa et al. (2017)	Egypt	Leaves	0, 5.2	Nzealand	8	Unknown	GP, H, BC
Etchu et al. (2017)	Cameroon	Leaves	0, 25, 50	Nzealand	7	Unsexed	H, BC
Aljohani and Abduljawad (2018)	S. Arabia	Leaves	0, 0.5, 1.0	Nzealand	8	Unknown	GP, H, BC
El‐Desoky et al. (2018)	Egypt	Leaves	0, 3, 6	Nzealand	10	Unsexed	GP, CT
Omara et al. (2018)	Egypt	Leaves	0, 10, 20, 30	Nzealand	12	Male	GP, H, BC
Sun et al. (2018)	China	Leaves	0, 10, 20, 30	Nzealand	< 8	Unknown	GP, BC
Singer et al. (2018)	Egypt	Leaves	0, 25, 50, 75	Nzealand	8	Unknown	GP, H, BC
Elkloub et al. (2018)	Egypt	Leaves	0, 0.25, 0.50, 0.75	Californian	8	Unsexed	GP, BC
Ayandiran et al. (2019)	Nigeria	Leaves	0, 25, 50, 100	Mixed	8	Unknown	H, BC
Jiwuba and Ogbuewu (2019)	Nigeria	Leaves	0, 10, 20, 30	Mixed	< 8	Unknown	GP, CT
Khalil et al. (2019)	Egypt	Leaves	0, 1, 2, 3	Nzealand	8	Male	H, BC
Ogunlade et al. (2019)	Nigeria	Leaves	0, 5, 10, 15	Mixed	> 12	Male	GP, H, BC
Bakr et al. (2019)	Egypt	Leaves	0, 3, 6	Nzealand	8	Unsexed	GP, CT, H, BC
Zendrato et al. (2019)	Indonesia	Leaves	0, 20, 40, 60	Nzealand	8	Unknown	GP
Saka et al. (2019)	Nigeria	S. powder	0, 0.5,1, 2	Mixed	10	Male	GP, H, BC
Ndofor‐Foleng et al. (2019)	Nigeria	Leaves	0, 10, 20, 30	Crossbreed	12	Unknown	GP, H
El‐Adawy et al. (2020)	Egypt	Leaves	0, 1, 1.5	Nzealand	12	Male	GP, H
Hernández‐Fuentes et al. (2020)	Egypt	Leaves	0, 10, 20, 30	Crossbreed	10	Unknown	GP, CT, H
Egu (2021)	Nigeria	Leaves	0, 5, 10.5, 15	Nzealand	8	Male	GP
Selim et al. (2021)	Egypt	Leaves	0.5, 1.0, 1.5	Nzealand	< 8	Unsexed	GP, CT, BC
Mankga et al. (2022)	S. Africa	Leaves	0, 5, 10, 15	Nzealand	12	Male	GP, BC, H
El‐kashef (2022)	Egypt	Leaves	2.5, 5, 7.5	Nzealand	18	Unsexed	GP, BC, CT
Salem et al. (2022)	Egypt	Leaves	0, 10, 20, 30	Alexandria	5	Unsexed	BC, H
Alatrony et al. (2022)	Nigeria	Leaves	0, 1	Moshtoher	10	Male	GP
Rahmy et al. (2023)	Egypt	Leaves	0, 1, 2, 3	Nzealand	> 12	Unknown	GP, CT, BC, H
Bhatt et al. (2023)	India	Leaves	0, 70, 95	Chinchilla	8	Male	GP, CT

Abbreviations: BC, blood chemistry; CT, carcass traits; GP, growth performance; H, hematology; SD, study periods.

### 2.3 Data Analysis

OpenMEE software (Wallace et al. 2016) was used to analyze the data, which is built in R‐software. The MOLM effects on the growth performance of rabbits were assessed using a restricted maximum likelihood (REML) random effects model. The choice of the REM was based on the assumptions that the data included in the meta‐analysis were not identical; therefore, variance must be divided into within–studies and between–studies variance plus sampling error (Borenstein et al. 2009). The impact of MOLM inclusion on FI, average daily gain (ADG) and FCR was assessed using standard mean difference (SMD) (95% CI). The mean pooled result is deemed significant when the lower and upper CIs exclude 0. The points to the left of the line of no effect (SMD = 0) depict a reduction in the values of FI, FCR, and ADG, and the points to the right depict an increase in these values. The Cochran's Q test and Higgins and Thompson's *I* ^2^ statistic were used to assess heterogeneity (Higgins and Thompson 2002). The meta‐regression test was performed to determine the percentage of heterogeneity explained by analyzed moderators with < 10 studies due to low statistical power (Borenstein et al. 2009). Publication bias was carried out to ascertain the validity of the results of the meta‐analysis using Rosenberg's fail‐safe number (Nfs) (Rosenburg 2005). Pooled results were considered robust despite the evidence of publication bias if Nfs > 5*n* + 10, where *n* = dataset (Jennions et al. 2013).

## 3 Results

### 3.1 Feed Intake

**FIGURE 2:**
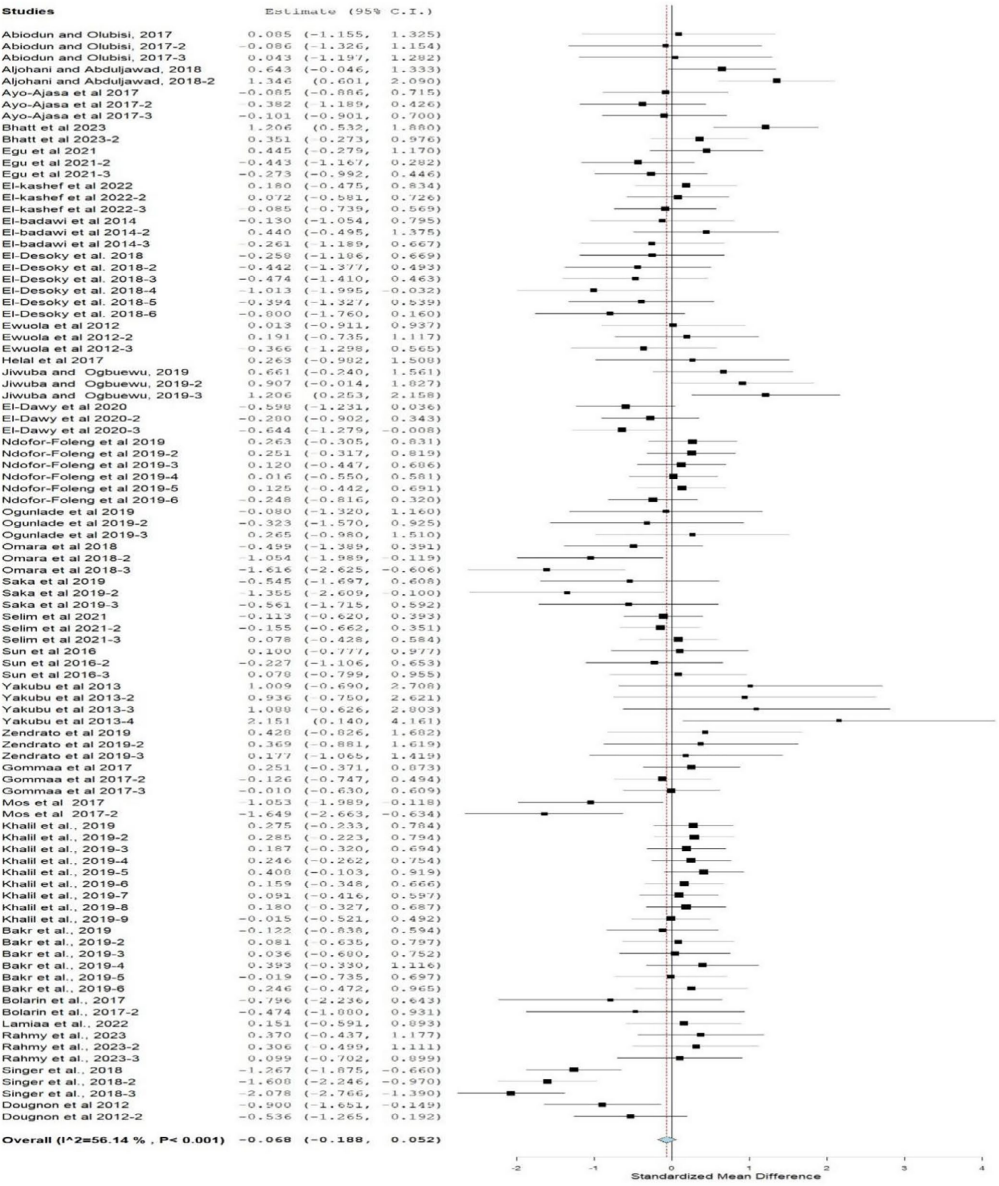
Forest plot of feed intake of rabbits fed diets containing *Moringa oleifera* leaf meal.CI = confidence interval; *I* ^2^ = inconsistency index, which measures the variance above chance among articles utilized in the analysis. The solid vertical line = mean difference of zero (0) or no effect. Points to the left of the zero = a decrease in FI, and to the right depict an increase in FI. Black square in the plot = the mean effect size for each experiment. The line joining the black squares = upper and lower 95% CI for the effect size. The dotted line with the diamond at the base showing 95% CI depicts the pooled estimation (considered significant when the line of 0 does not touch the diamond at the bottom of the forest plot).

A forest plot (Figure 2) shows that FI was not significantly affected by feeding rabbits a diet containing MOLM (SMD = −0.07; 95% CI: −0.19, −0.05; *p* = 0.05; *I* ^2^ = 56.1%). Subgroup meta‐analysis by inclusion levels authenticated that 0–5 had been significantly increased by MOLM inclusion only (Table 3). When compared to the control diet, a significantly lower FI value was observed at the 6%–10% inclusion levels. However, similar FI values were observed at 11%–15%, 16%–20%, and > 20% inclusion levels. Crossbreed subgroups had lower FI values than those fed the control diet, while FI values were not affected in other breeds. FI value was also not significantly affected by the sex subgroup (male, unknown, unsexed). Regarding subgroup feeding duration, reduced FI was observed in rabbits fed a MOLM‐containing diet for the duration of 10 and 12 weeks, but FI was not affected in other ages when compared to those fed the control diet. This study showed moderate heterogeneity in FI (*p* = < 0.001, *I* ^2^ = 56.1%; Figure 2) regarding the effects of feeding rabbits diets containing MOLM. Inclusion levels (%), rabbit breed, sex, and age in weeks were used as modifiers to explore the originality of this meta‐analysis as shown in Table 6. There is a significant relationship between modifiers (breeds) and FI in rabbits fed a MOLM‐based diet (MOLMBD).

**TABLE 3:** Effect of diet containing *Moringa oleifera* leaf meal on feed intake of rabbits.

Subgroup	Nc	Random effects			Heterogeneity	
		SMD	95%, CI	*p*	*I* ^2^ (%)	*p*
Inclusion (%)						
0–5	38	0.12	0.11, 0.23	0.03	0.0	0.69
6–10	22	0.27	−0.49, −0.05	0.02	40.9	0.03
11–15	7	−0.13	−0.52, 0.26	0.51	21.9	0.26
16–20	4	−0.12	−0.63, 0.38	0.63	50.2	0.11
> 20	23	−0.05	−0.45, 0.34	0.79	80.5	0.00
Breed						
Crossbreed	13	−0.32	−0.60, −0.04	0.03	32.7	0.12
New Zealand	62	−0.09	−0.23, 0.05	0.19	58.1	0.00
Not stated	3	−0.19	−0.65, 0.28	0.43	0.0	0.85
Chinchilla	2	0.77	−0.07, 1.61	0.07	69.9	0.07
Mixed breeds	13	0.33	−0.15, 0.81	0.18	48.3	0.03
Monshtoher	0	0.15	−0.59, 0.89	NA	NA	NA
Sex						
Male	34	0.03	−0.20, 0.14	0.76	41.5	0.01
Unsexed	34	0.01	−0.25, 0.95	0.95	27.6	0.01
Unknown	26	−0.17	−0.37, 0.03	0.09	72.0	0.00
Feeding duration (weeks)						
< 8	22	0.08	−0.07, 0.23	0.28	6.8	0.37
8	36	−0.01	−0.27, 0.25	0.92	75.6	0.00
10	13	0.30	−0.55, −0.05	0.02	0.0	0.60
12	14	−0.27	−052, −0.01	0.04	44.7	0.00
> 12	9	0.13	−0.21, 0.48	0.45	0.0	1.00

Abbreviations: CI, confidence interval; *I* ^2^, inconsistency index; Nc, number of comparisons; *p*, probability difference; SMD, standardized mean difference.

### 3.2 Weight Gain

**FIGURE 3:**
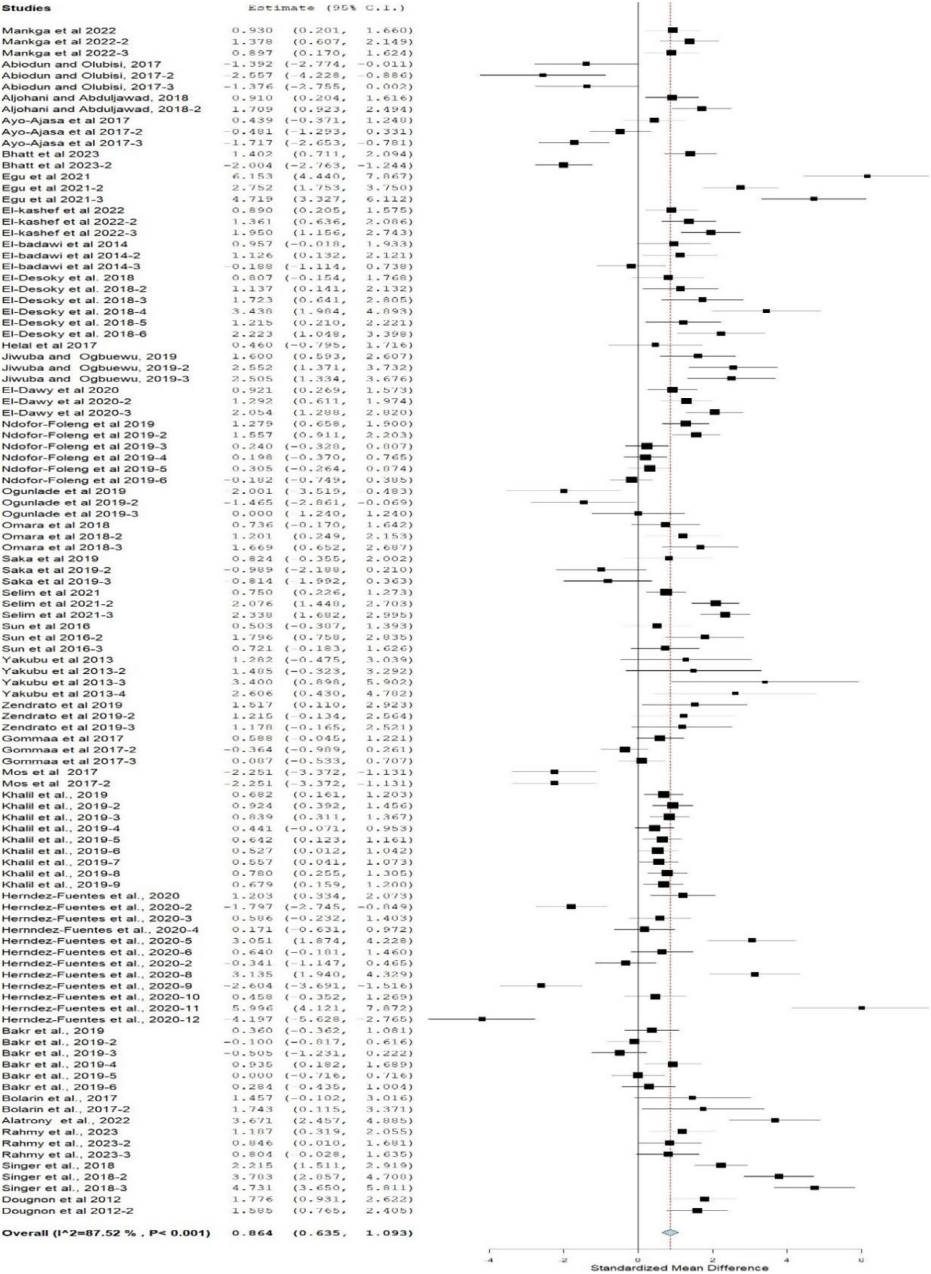
Forest plot of weight gain of rabbits fed diets containing *Moringa oleifera* leaf meal. CI = confidence interval; *I* ^2^ = inconsistency index which measures the variance above chance among articles utilized in the analysis. The solid vertical line = mean difference of zero (0) or no effect. Points to the left of the zero = a decrease in FI, and to the right depicts an increase in FI. Black square in the plot = the mean effect size for each experiment. The line joining the black squares = upper and lower 95% CI for the effect size. The dotted line with a diamond at the base showing the 95% CI.

A forest plot (Figure 3) shows the influence of MOLM when incorporated in the diet of rabbits, on average daily gain (ADG). In this Figure, MOLMBDs significantly increased ADG (SMD = 0.86; 95% CI: 0.64, 0.09; *p* < 0.001; *I* ^2^ = 87.5%) in rabbits, which is influenced by modifiers as described in Table 4. The stratified analysis authenticates that inclusion levels and sex significantly improved weight gain compared to those reared on the control diet. ADG was not affected in crossbreed, chinchilla, unknown, and mixed breeds reared on MOLM‐containing diets compared with those reared on the control diet, whereas it increased in the New Zealand breed (*p* < 0.05). Rabbits fed MOLM for < 8, 8, and 12 weeks significantly increased ADG, while those fed for 10 and 12 weeks showed a significantly similar ADG. Table 6 shows a relationship between ADG and modifiers (breed only).

**TABLE 4:** Effect of diet containing *Moringa oleifera* leaf meal on weight gain of rabbits.

Subgroup	Nc	Random effects			Heterogeneity	
		SMD	95%, CI	*p*	*I* ^2^ (%)	*p*
Inclusion (%)						
0–5	41	0.83	0.56, 1.09	< 0.001	80.3	0.00
6–10	23	0.63	0.15, 1.11	0.01	85.6	0.00
11–15	7	1.60	0.63, 2.58	0.001	84.1	0.00
16–20	8	1.78	0.58, 2.99	0.004	92.6	0.00
> 20	27	0.75	0.13, 1.38	0.02	92.1	0.00
Breed						
Crossbreed	22	0.004	−0.67, 068	1.00	91.7	00
New Zealand	65	1.20	1.00, 1.41	< 0.001	81.2	00
Not stated	3	−0.57	−1.75, 0.62	0.35	82.8	0.003
Chinchilla	2	−0.30	−3.64, 3.04	0.86	97.6	00
Mixed breeds	13	0.76	−0.15, 1.66	0.10	82.1	00
Monshtoher	0	3.67	2.6, 4.89	NA	NA	NA
Sex						
Male	37	0.73	0.37, 1.08	< 0.001	96.3	00
Unsexed	26	0.75	0.25, 1.25	0.004	86.7	00
Unknown	43	1.06	0.68, 1.44	< 0.001	89.2	00
Feeding duration (weeks)						
< 8	12	1.52	1.08, 1.96	< 0.001	67.3	00
8	43	1.00	0.65, 1.35	< 0.001	89.0	00
10	25	0.67	−0.1, 1.35	0.05	91.4	00
12	17	0.96	0.64, 1.29	< 0.001	68.0	00
> 12	12	−053	−1.42, 0.36	0.24	81.1	00

Abbreviations: CI, confidence interval; *I* ^2^, inconsistency index; Nc, number of comparisons; *p*, probability difference; SMD, standardized mean differences.

### 3.3 Feed Conversion Ratio

**FIGURE 4:**
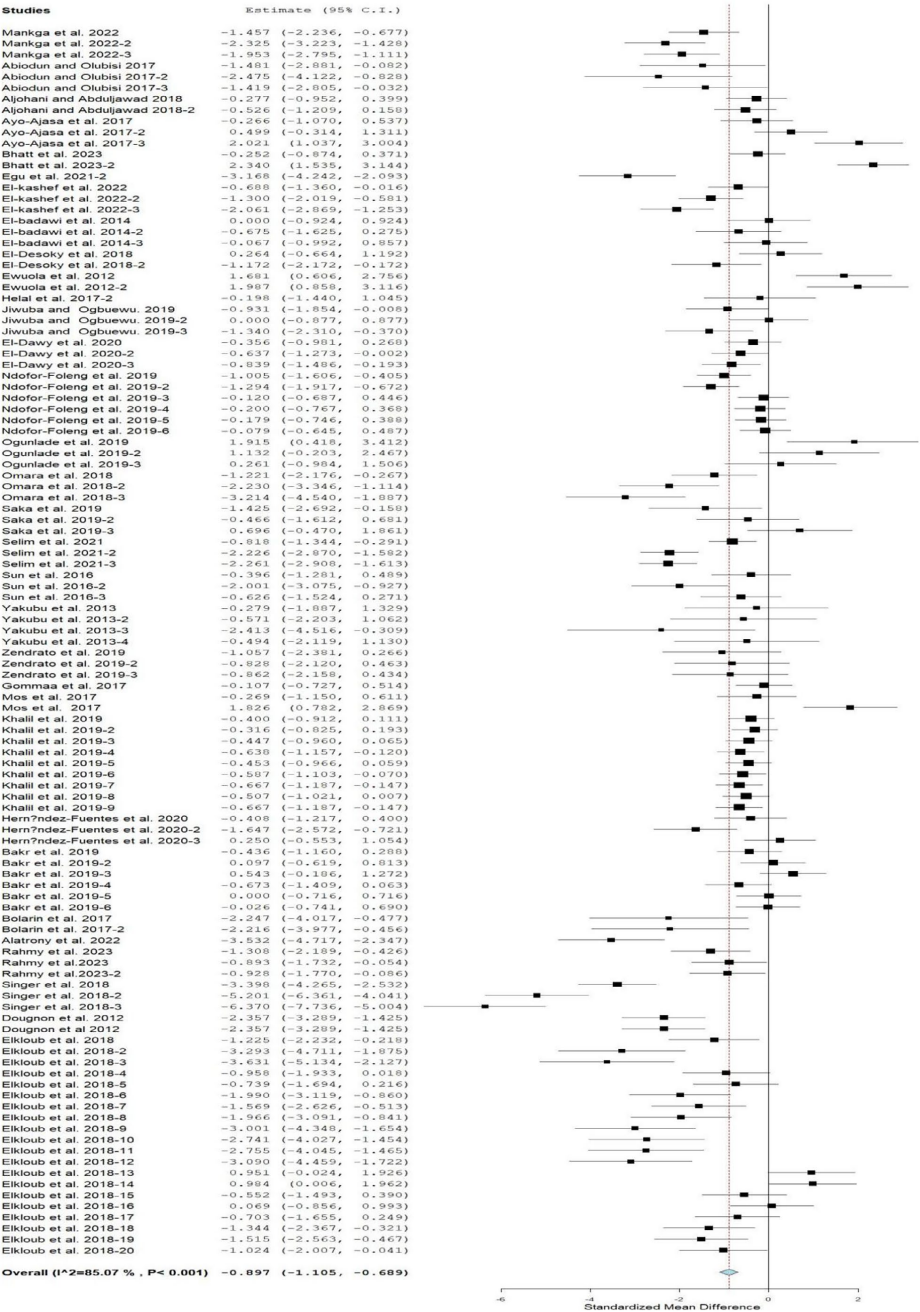
Forest plot of FCR of rabbits fed diets containing *Moringa oleifera* leaf meal. CI = confidence interval; *I* ^2^ = inconsistency index, which measures the variance above chance among articles utilized in the analysis. The solid vertical line = mean difference of zero (0) or no effect. Points to the left of the zero = a decrease in FI, and to the right depict an increase in FI. Black square in the plot = the mean effect size for each experiment. Line that joined the black squares = upper and lower 95% CI for the effect size. The dotted line with the diamond at the base showing the 95% CI depicts the pooled estimation (considered significant when the line of 0 does not touch the diamond at the bottom of the forest plot).

The pooled effect of MOLM inclusion in diets fed to rabbits on FCR is shown in Figure 4. The results reveal that MOLM inclusion in rabbit diets promoted FCR (SMD = 0.89; 95% CI = −1.11, −0.68; *p* < 0.001). Table 5 illustrates the effects of MOLM inclusion in rabbit diets on FCR. FCR was promoted in all inclusion levels and sexes compared with those fed the control diet. Crossbreed, not stated, chinchilla, and mixed breeds had a similar FCR to those fed the control diet. Interestingly, the New Zealand breed had the best FCR by a significant margin when fed a MOLMBD. Rabbits reared on diets containing MOLM for < 8, 8, and 12 weeks had the best FCR, but those reared on MOLM diets for 10 and 12 weeks had a similar FCR to those on the control diet. Heterogeneity exists among the trials used for the investigation (*p* < 0.001, *I* ^2^ = 85.1%; Figure 4). Table 6 shows a relationship between FCR and modifiers (breed only).

**TABLE 5:** Effect of diet containing *Moringa oleifera* leaf meal on FCR of rabbits.

Subgroup	Nc	Random effects			Heterogeneity	
		SMD	95%, CI	*p*	*I* ^2^ (%)	*p*
Inclusion (%)						
0–5	56	−0.88	−1.11, −0.64	< 0.001	79.0	00
6–10	22	−0.67	−1.10, −0.24	0.002	83.3	00
11–15	6	−1.05	−2.01, −0.10	0.03	81.6	00
16–20	5	−1.39	−2.17, −0.61	< 0.001	78.4	0.001
> 20	22	−1.05	−1.81, −0.29	0.006	92.8	00
Breed						
Crossbreed	15	−0.44	−1.05, 0.18	0.17	86.7	00
New Zealand	57	−1.06	−1.31, −0.82	< 0.001	83.8	00
Not stated	3	0.72	−0.53, 1.97	0.26	84.1	0.002
Chinchilla	2	1.03	−1.51, 3.57	0.43	96.0	0.000
Mixed breeds	13	−0.27	−0.83, 0.29	0.34	60.3	0.003
Monshtoher	0	−3.53	−4.72, −2.35	NA	NA	NA
Sex						
Male	35	−0.76	−1.07, −0.44	< 0.001	82.3	00
Unsexed	42	−0.90	−1.28, −0.53	< 0.001	84.0	00
Unknown	34	−1.04	−1.45, −0.64	< 0.001	88.4	00
< 8	26	−1.31	−1.76, −0.86	< 0.001	82.4	00
Feeding duration (weeks)						
8	43	−0.78	−1.10, −0.46	< 0.001	86.8	00
10	16	−0.42	−1.16, 0.33	0.28	89.1	00
12	17	−1.10	−1.49, −0.72	< 0.001	77.6	00
> 12	8	−0.59	−1.35, 0.16	0.12	73.6	00

Abbreviations: CI, confidence interval; *I* ^2^, inconsistency index; Nc, number of comparisons; *p*, probability difference; SMD, standardized mean differences.

**TABLE 6:** Meta‐regression comparing the associations between moderators and measured outcomes.

Outcomes	Moderators	Intercept	*Q*_M_	Estimate	df	*p*	*R* ^2^ (%)
Feed intake (g/rabbit/day)	Breed	−0.33	11.23	0.17	5	0.05	9.85
	Duration	0.11	6.04	0.19	4	0.20	1.35
	Inclusion	0.09	6.13	0.18	4	0.19	6.92
	Sex	−0.05	1.01	0.20	2	0.60	0.00
Weight gain (g/rabbit/day)	Breed	1.24	20.87	1.77	5	< 0.001	16.00
	Duration	1.02	11.38	1.97	4	0.02	6.60
	Inclusion	0.81	5.03	2.11	4	0.29	0.18
	Sex	0.72	1.17	2.13	2	0.56	0.00
Feed conversion ratio	Breed	−1.09	25.02	1.26	6	< 0.001	17.47
	Duration	−1.19	6.30	1.50	4	0.18	17.77
	Inclusion	−0.91	1.72	1.56	4	0.79	0.00
	Sex	−0.77	0.74	1.55	2	0.69	0.00

Abbreviations: df, degree of freedom; *p*, probability difference, *Q*_M_, coefficient of moderator; *R* ^2^, amount of heterogeneity explained by moderators.

### 3.4 Analysis of Publication Bias

**FIGURE 5:**
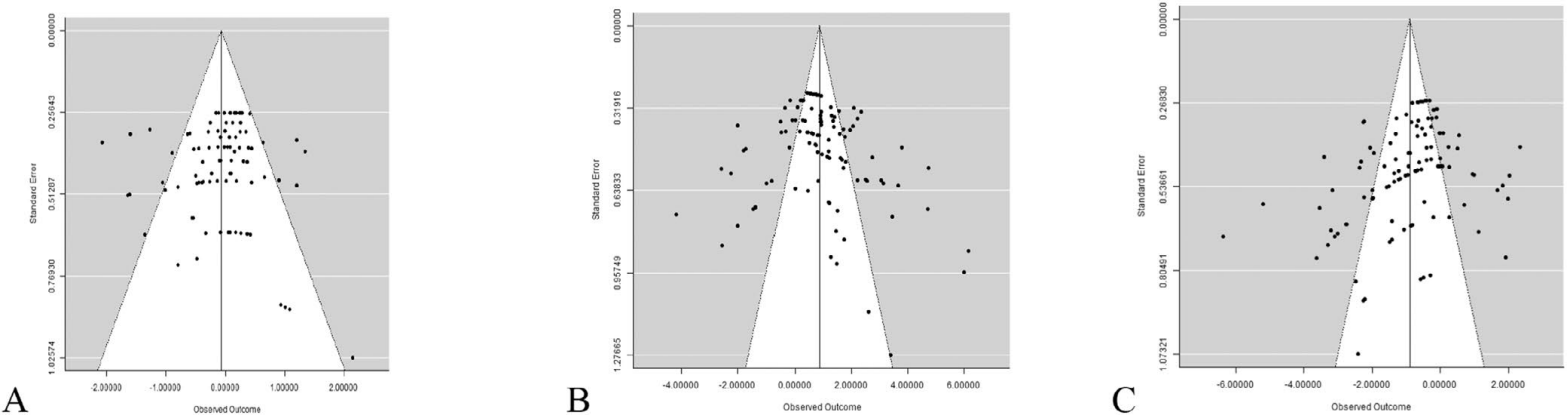
Funnel graphs of the effect of *Moringa oleifera* leaf meal‐containing diet fed to rabbits on (A) weekly fed intake, (B) average daily gain, and (C) feed conversion ratio.

Funnel graphs (Figure 5) show a weak tendency for smaller studies to be associated with greater negative effects. The funnel graphs produced were symmetrical and Rosenberg's Nfs was also employed to check for evidence of publication bias. The Nfs for the database are 33 (FI), 616 (FCR), and 387 (ADG) which were 0.3‐, 5‐, and 3‐fold higher than the thresholds of 115 (5 × 21 + 10), 115 (5 × 21 + 10), and 130 (5 × 24 + 10), respectively; therefore, declaration of the robustness of the mean effect size was required.

### 3.5 Hematology of Rabbit Fed MOLM‐Based Diets

This meta‐analysis revealed a lack of treatment effects on hemoglobin, MCHC, PCV, and neutrophils in rabbits fed MOLMBD (Table 7). A reduction effect (*p* < 0.05) was observed on blood MCV, MCH, and lymphocyte contents in rabbits fed MOLMBD. Interestingly, WBC and RBC contents improved significantly in rabbits fed a MOLMBD.

**TABLE 7:** Effects of *Moringa oleifera* leaf meal on hematological parameters of rabbits in % unless otherwise stated.

Parameters	SMD	95% Cl		SE	*p*	Heterogeneity			
		Lower	Upper			*Q*_M_	df	*I* ^2^ (%)	*p*
Hemoglobin	0.17	−0.03	0.37	0.01	0.09	76.0	32	58.0	< 0.001
MCV, Pg	−0.29	−0.50	−0.06	0.01	0.005	17.4	19	0.0	0.57
MCH, Pg	−0.35	−0.56	−0.13	0.11	0.002	11.8	7	0.0	0.81
MCHC	0.02	−0.19	0.22	0.10	0.87	7.0	19	0.0	0.99
PCV	0.19	−0.14	0.51	0.16	0.26	168.1	31	81.6	< 0.001
WBC, U/L	0.93	0.44	1.41	0.25	< 0.001	401.4	36	91.0	< 0.001
RBC, U/L	0.68	0.36	0.99	0.16	< 0.001	233.5	38	83.7	< 0.001
Lymphocytes	−0.97	−1.47	−0.47	0.26	< 0.001	92.3	19	79.4	< 0.001
Neutrophils	0.17	−0.47	0.80	0.33	0.61	104.8	16	84.7	< 0.001

Abbreviations: CI, confidence interval; df, degree of freedom; *l* ^2^, inconsistency index; MCH, mean corpuscular hemoglobin; MCHC, mean corpuscular hemoglobin concentration; MCV, mean corpuscular volume; *p*, probability difference; PCV, packed cell volume; Pg, picograms; *Q*_M_, coefficient of moderator; RBC, red blood cell; SE, standard error; SMD, standardized mean difference; U/L, units per liter; WBC, white blood cell.

### 3.6 Blood Plasma Profile and Carcass Characteristics of Rabbits Fed MOLM‐Based Diets

Meta‐analysis results of blood chemistry of rabbits fed MOLMBD (Table 8) show that total protein, albumen, and globulin improved in rabbits fed treatment diets compared to those in the control group. There was no significant difference in glucose and alanine aminotransferase (ALT) between rabbits in the control and the treatment groups. However, triglycerides, cholesterol, urea, creatinine, and aspartate aminotransferase (AST) contents decreased (*p* > 0.05) in rabbits fed a diet containing MOLM compared to those fed controls.

**TABLE 8:** Effects of *Moringa oleifera* leaf meal on plasma profiling of rabbit.

Parameters	SMD	95% Cl		SE	*p*	Heterogeneity			
		Lower	Upper			*Q*_M_	df	*I* ^2^(%)	*p*
Plasma protein profile									
Total protein	0.40	0.10	0.71	0.16	0.009	357.3	49	86.3	< 0.001
Albumen	0.43	0.14	0.71	0.15	0.003	374.3	50	86.6	< 0.001
Globulin	0.59	0.15	1.03	0.22	0.009	536.8	44	91.8	< 0.001
Urea	−0.94	−0.73	−0.25	0.12	< 0.001	86.3	24	72.2	< 0.001
Creatinine	−129	−1.65	−0.92	0.19	< 0.001	182.5	27	85.2	< 0.001
Plasma energy profile									
Triglycerides	−1.37	−1.92	−0.83	0.28	< 0.001	213.6	17	92.0	< 0.001
Glucose	0.03	−0.60	0.66	0.32	0.92	113.8	13	88.6	0.001
Cholesterol	−0.85	−1.24	−0.46	0.20	< 0.001	522.2	45	19.4	< 0.001
Plasma enzyme profile (IU/L)									
Aspartate aminotransferase	−0.41	−0.69	−0.12	0.15	0.005	164.1	32	80.5	< 0.001
Alanine aminotransferase	0.03	−0.22	0.29	0.13	0.80	305.9	45	85.3	< 0.001

Abbreviations: CI, confidence interval; df, degree of freedom; *l* ^2^ , inconsistency index; *p*, probability difference; *Q*_M_, coefficient of moderator; SE, standard error; SMD, standardized mean difference.

Meta‐analysis results show that treated diets had higher carcass yield and dressing percentage compared to the control diet (Table 9). No treatment effects (*p* < 0.05) were observed on abdominal fats.

**TABLE 9:** Effects of *Moringa oleifera* leaf meal on carcass characteristics of rabbit.

Variables	SMD	95% Cl		SE	*p*	Heterogeneity			
		Lower	Upper			*Q*_M_	df	*I* ^2^(%)	*p*
Carcass yield	1.10	0.66	1.55	0.23	< 0.001	331.5	32	90.3	< 0.001
Dressing %	0.32	0.18	0.46	0.07	< 0.001	65.7	40	39.1	0.006
Abdominal fats	−0.81	−2.01	0.46	0.65	0.21	109.9	7	93.6	< 0.001

Abbreviations: CI, confidence interval; df, degree of freedom; *I* ^2^, inconsistency index; *p*, probability difference; *Q*_M_, coefficient of moderator; SE, standard error; SMD, standardized mean difference.

## 4 Discussion

### 4.1 Feed Intake

In this study, we sought to evaluate in a meta‐analysis the effects of feeding rabbits a diet containing MOLM. MOLM is known to be rich in phenylalanine, lysine, leucine, and other essential acids (Natsir et al. 2019), which are responsible for growth performance and production of antibodies (Bunchasak 2009). It also contains trace minerals (Sebola et al. 2019; Valdez‐Solana et al. 2015) as a component of enzymes, hormones, vitamins, and as cofactors in metabolism, catalysts, and enzyme activators. Regardless of high nutritional values, their level of intake could be restricted by the presence of excessive fiber and ANFs, which can significantly affect the palatability and digestibility of the diet. Additionally, raw material in the diets influences taste, palatability, and functional properties, leading to alteration in FI (Disetlhe et al. 2018). The literature (Sun et al. 2018; El‐kashef 2022; Bhatt et al. 2023) reports that MOLM inclusion in the rabbit diet did not significantly affect their FI, which supports the results of this current meta‐analysis. Similarly, FI in rabbits fed a MOLMBD and those fed the control diet suggests that MOLM inclusion did not affect the palatability of the diet. Conversely, Khalid et al. (2020) found that feeding dietary MOLM to stressed rabbits increased FI. Nevertheless, it appears that the ANFs, such as condensed tannin and cellulose that are present in MOLM, did not reduce FI in the rabbits. Indeed, Mankga et al. (2022) reported high fiber digestibility of 27.6% in rabbits fed 15% MOLM compared to 21.6% in those fed the control diet.

Rabbits fed diets containing 0%–5% MOLM had higher FI, suggesting that they did not face any difficulties in consuming and utilizing the feed efficiently. This observation could be associated with low MOLM inclusion, which could result in reduced toxicity and moderate fiber intake. This is consistent with the findings of Jiwuba and Ogbuewu (2019), who reported high FI of rabbits when fed a diet containing 10% MOLM inclusion. Rabbits fed diets containing 11%–15%, 11%–20%, and > 20% MOLM had a comparable FI to those fed the control diet, suggesting that MOLM can be incorporated in rabbits' diets evenly at a high percentage. These results are in line with those of Ayandiran et al. (2019) and Ghomsi et al. (2017) when MOLM was included at 100% and 50%, respectively, in the rabbit diet. The finding of this meta‐analysis shows that rabbit breeds are sources of heterogeneity in trials that explored the impact of MOLM on rabbits' FI. Crossbreeds had a decreased FI, indicating their inability to overcome ANFs that reduce the palatability in the diet (tannin). Several studies have indicated that high tannin content has negative effects on FI, palatability, and digestibility of nutrients due to its astringent taste (Hassan et al. 2003; Makkar 2003; Kim and Miller 2005) and results in decreases in weight gain. The comparable FI in New Zealand, not stated, mixed, and Monshtoher breeds with controls suggests their ability to overcome fiber and ANF content in the diets. This agrees with Zendrato et al. (2019) and Sun et al. (2018), who fed rabbits diets containing MOLM at 10, 20, 40, 60 and 10, 20, 30, respectively. In this meta‐analysis, FI was not affected by subgroup sex, implying similar taste buds and gut microbiota; this is in line with Salisu and Iyeghe‐Erakpotobor (2014), who reported that FI of rabbits was not affected by sex. The pooled effect shows that rabbits fed diets containing MOLM for < 8, 8, and > 12 weeks had similar FI, suggesting that they developed adaptation mechanisms to overcome condensed tannin and cellulose. Conversely, rabbits fed diets containing MOLM for 10 and 12 weeks had a decreased FI, which is surprising because high FI was expected at long feeding duration. A similar result was obtained by El‐Desoky et al. (2018) in a study where rabbits were fed a MOLMBD for 18 weeks, although a lower inclusion level of 6% was used.

### 4.2 Weight Gain and FCR

*Moringa oleifera* leaves (MOL) are rich in high biological value protein and essential amino acids. Methionine and lysine are vital for muscle development (Fang et al. 2021). The best (*p* > 0.05) ADG and FCR in rabbits reared on MOLM‐containing diets imply that the rabbits were able to utilize nutrients in the amount of feed consumed, which could be linked to improved morphometric characteristics of the intestinal mucosa and gut microbiota. The superior ADG and FCR in rabbits fed diets containing MOLM suggest that these rabbits effectively utilized the nutrients from the feed they consumed. This improvement may be associated with enhanced morphometric characteristics of the intestinal mucosa and a healthier gut microbiota. Indeed, Ogbuewu and Mbajiorgu (2022) highlight the intestine as the principal site for immunity, nutrient digestion, and uptake in animals. These results are the same as those of Jiwuba and Ogbuewu (2019), who reported improved ADG and FCR when 30% MOLM was included in rabbit diets, and Zendrato et al. (2019) reported similar ADG when 60% MOLM was incorporated in rabbit diets. Abiodun and Olubisi (2017) and also reported lower ADG in rabbits fed 7.5% and 50% of MOLM inclusion, respectively. Nevertheless, it appears that this meta‐analysis resolves these discrepancies, and it can be concluded that MOLM inclusion in rabbit diets improves ADG and FCR. The results of this study show that ADG and FCR improve based on inclusion levels of up to > 20% and sex in comparison with the control, indicating that these inclusion levels are well‐tolerated. This corroborates with Sun et al. (2018) and El‐kashef (2022) when 10%, 20%, and 30% MOLM were included in rabbit diets. It is important to note that *Moringa oleifera* contains bioactive compounds (flavonoids, ascorbic acid, phenolic and carotenoid) that act as natural antioxidants, which could be linked to this improved ADG and FCR. The finding of the study shows that breeds are sources of heterogeneity when exploring the impact of MOLM on weight gain of rabbits. This meta‐analysis indicates that New Zealand breeds fed MOLM had improved ADG and FCR compared to the control, which corroborates Mankga et al. (2022), who reported higher ADG and FCR in the New Zealand breed fed a diet containing 15% MOLM. The improved ADG and FCR could be linked to breed differences, which indicates the capacity of the New Zealand breed to utilize MOLM‐containing diets. Further research is needed to investigate the variables responsible for improved FCR in the New Zealand breed when feeding MOLM to incorporate it in other breeds. The comparable ADG and FCR in crossbred, not‐stated, chinchilla, mixed breed, and Monshtoher with those in the control imply that the MOLM diets were fully converted into 1 g of muscle at the same rate as those in the control group. It can be postulated that the digestive capacity of different breeds differs when challenged with MOLM. The subgroup analysis showed that MOLM was well utilized by rabbits when fed for < 8, 8, and 12 weeks, as reflected by improved ADG and FCR, confirming a well‐developed gut during these feeding periods. Bivolarski and Vachkova (2014) conclude that a well‐developed anatomical structure and functioning of the small intestine fully facilitate feed absorption and utilization. The development of the intestine is directly related to the growth and development of rabbits (Liu et al. 2022). A similar ADG and FCR was recorded in rabbits fed a diet containing MOLM for 10 and 12 weeks, implying that the efficiency of feed with those on the control diet was similar. Such a feed ingredient can therefore be used in rabbit feeding to promote ADG and FCR. It can be postulated that rabbits possess a well‐developed gastrointestinal gut (pseudo ruminants) and good cellulose‐degrading gut microbiota (Khalid et al. 2020) that assist in utilizing fibrous diets and tolerating anti‐nutritional compounds such as tannin.

### 4.3 Hematology and Plasma Biochemistry

Blood parameters are a useful tool for assessing rabbits' health and nutritional status as well as other physiological disturbances in the environment. These parameters can be influenced by various factors including stress, hydration status, and other dietary components. Our meta‐analysis results show that MCV, MCH, and lymphocytes were lower in rabbits fed MOLMBDs compared to those fed control. However, it is known that lower MCV and MCH values could indicate changes in iron metabolism or utilization while lymphocyte variations may reflect immune system modulation. The lower FI in this study could have contributed to these results. Moreover, further research is needed to understand the physiological significance of these hematological changes and their potential relationship to nutrient metabolism.

The observed higher (*p* < 0.05) WBC and RBC in rabbits fed MOLMBD in comparison with control suggests that MOLM may contain metabolites, which may increase bone marrow functioning, in turn increasing leukopoiesis and erythropoiesis. Some researchers associate high WBC with microbial infections or the presence of antigens in blood, whereas higher RBC depicts disease‐free animals (Bolarin et al. 2017). The comparable hemoglobin, MCHC, PCV and neutrophil values in rabbits fed test diets with those fed control suggest that the amount of oxygen circulating in the body and the immune status were not negatively affected by feeding MOLMBD. These results are in line with the findings of Anthony and Ashawe (2014) and Ewuola et al. (2012), who found a lack of dietary effects on blood hematology of rabbits fed a diet containing 0%, 5%, 10%, and 15% MOLM. The improvement in total protein, albumin, and globulin in rabbits offered MOLMBD in comparison with control indicates the ability of MOLM to release its bioactive compounds during digestion, resulting in an improvement in blood protein and is confirmed by Ogbuewu and Mbajiorgu (2023) when MOLM was used in broiler chickens.

Therefore, it can be deduced that MOL contains bioactive compounds (Sebola et al. 2015) that promote other blood parameters; this is also reflected by improved weight gain. These results are consistent with the findings of Selim et al. (2021), who reported improved blood protein and globulin in rabbits fed a diet containing 1.5% MOLM inclusion. Additionally, Jiwuba et al. (2016) also reported similar results when rabbits were fed a diet containing 0%, 10%, 20%, 30% of MOLM. The observed significantly lower urea, creatinine, and triglycerides in rabbits offered MOLMBD compared to the control imply that feeding a diet containing MOLM to rabbits can reduce urea, creatinine, and triglycerides levels in the meat. Lower urea suggests that the test diet improved the functioning of kidneys, and the lower blood creatinine and triglycerides indicate low energy supply from the cell to the muscle. This may be attributable to low dietary fat supplied by MOLMBD, which would subsequently be evident in the blood. Contrary to the current results, Yakubu et al. (2013) observed similar serum biochemical indices in rabbits fed a diet containing 0%, 25%, 50%, 75%, and 100% MOLM. Blood glucose, cholesterol, and abdominal fats are indicators of energetic and lipid metabolism, but in this meta‐analysis these were not affected by MOLM inclusion, as shown by their similar contents. This could be due to high‐quality protein present in MOLM (El‐Desoky et al. 2018). The comparable plasma enzymes (indicators of the integrity of vital organs) in rabbits fed test diets compared to the control indicate that any anti‐nutritional factor present in MOLM was not systemically harmful to the rabbits. It is known that ingestion of glucosinolates present in canola can lead to reduced FI and liver damage (elevated plasma enzymes) (Campbell and Slominski 1991). Similar results were observed by Bakr et al. (2019), who reported a lack of significant difference in glucose and cholesterol in rabbits fed 3% and 6% MOLM inclusion. Evidence exists that MOLM is rich in essential amino acids (lysine and methionine) and bioactive compounds (quercetin), both of which are responsible for muscle development (Fang et al. 2021). This could be associated with the high carcass yield and dressing percentage observed in rabbits offered a MOLMBD. Carcass yield in rabbits is a reliable indicator of diet quality. This meta‐analysis indicates that MOLM improves edible meat when incorporated in the diets of rabbits. Similar observations were reported by Mankga et al. (2022) and Bhatt et al. (2023), who found higher slaughter and carcass weight in rabbits fed a MOLMBD. Rahmy et al. (2023) report a higher dressing percentage in rabbits fed 0, 10, 30, and 40 g/kg of MOLM. It can be deduced that the less expensive MOLM can be included in rabbit diets without compromising their carcass yield.

### 4.4 Limitations and Strengths of the Meta‐Analysis

The effect of MOLMBD on the performance of rabbits was assessed; however, this finding may apply to rabbits only. Inclusion levels, strain, processing methods, and duration of feeding influence the findings of this meta‐analysis. Once again, studies included in the meta‐analysis may have employed different analytical techniques, which could be a limitation, and other hematological parameters on rabbits fed MOLMBDs were not analyzed due to insufficient data. On the contrary, this meta‐analysis was the first study to summarize all published evidence on the effects of feeding rabbits a diet containing MOLM on growth, hemo‐biochemical parameters, and slaughter performance, which will provide a crucial role in the use of MOLM in rabbit nutrition. Also, it has established the standards for measuring and reporting MOLM effects on rabbits in the future.

## 5 Conclusions and Future Research Directions

The meta‐analysis results show that feeding rabbits a diet containing MOLM did not significantly affect FI, but it improved ADG and FCR. However, subgroup analysis reveals that 0%–5% MOLM inclusion had a significantly higher FI. Crossbred breed and feeding MOLM for 10 and 12 weeks significantly lower FI. However, FI was not affected by sex in the rabbits fed a diet containing MOLM. Pooled results indicate that feeding rabbits a diet containing MOLM improves weight gain. Weight gain was improved in all the inclusion levels, New Zealand breed, and sex and feed periods compared to the control diet. Subgroup of inclusion levels and sex had showed to improve FCR in rabbits fed a MOLMBD in this meta‐analysis. Hemo‐biochemical parameters and slaughter performances of rabbit were positively affected by MOLMBD. It can be concluded that feeding rabbits a diet containing MOLM improves growth performance, hemo‐biochemical parameters, and slaughter performances without adverse effects. Future research directions on the effects of MOLM on rabbit performance using specific search, rather than broad search terms (e.g., “feed conversion ratio,” “average daily weight gain”) are needed.

## Author Contributions

**Freddy Manyeula:** conceptualization (equal); data curation (supporting), formal analysis (supporting), investigation (equal), methodology (supporting), project administration (equal), supervision (equal), validation (supporting), visualization (supporting), writing – review and editing (equal). **Nthabiseng Amenda Sebola:** conceptualization (equal), data curation (lead), formal analysis (lead), investigation (equal), methodology (lead), project administration (equal), supervision (equal), validation (equal), visualization (lead), writing – original draft (lead), writing – review and editing (equal). **Monnye Mabelebele:** conceptualization (equal), data curation (lead), formal analysis (lead), investigation (equal), methodology (lead), project administration (equal), supervision (equal), validation (equal), visualization (lead), writing – original draft (lead), writing – review and editing (equal).

## Conflicts of Interest

The authors declare no conflicts of interest.

## Data Availability

The data that support the findings of this study are available on request from the corresponding author.
